# Maternal pre-pregnancy body mass index and foetal acidosis in vaginal and caesarean deliveries: The Japan Environment and Children’s Study

**DOI:** 10.1038/s41598-020-79178-1

**Published:** 2021-02-23

**Authors:** Tsuyoshi Murata, Hyo Kyozuka, Akiko Yamaguchi, Toma Fukuda, Shun Yasuda, Akiko Sato, Yuka Ogata, Kosei Shinoki, Mitsuaki Hosoya, Seiji Yasumura, Koichi Hashimoto, Hidekazu Nishigori, Keiya Fujimori, The Japan Environment and Children’s Study (JECS) Group, Michihiro Kamijima, Shin Yamazaki, Yukihiro Ohya, Reiko Kishi, Nobuo Yaegashi, Chisato Mori, Shuichi Ito, Zentaro Yamagata, Hidekuni Inadera, Takeo Nakayama, Hiroyasu Iso, Masayuki Shima, Youichi Kurozawa, Narufumi Suganuma, Koichi Kusuhara, Takahiko Katoh

**Affiliations:** 1 Fukushima Regional Center for the Japan Environmental and Children’s Study, 1 Hikarigaoka, Fukushima, 960-1295 Japan; 2 Department of Obstetrics and Gynecology, Fukushima Medical University School of Medicine, 1 Hikarigaoka, Fukushima, 960-1295 Japan; 3 Department of Pediatrics, Fukushima Medical University School of Medicine, 1 Hikarigaoka, Fukushima, 960-1295 Japan; 4 Department of Public Health, Fukushima Medical University School of Medicine, 1 Hikarigaoka, Fukushima, 960-1295 Japan; 5 Fukushima Medical Center for Children and Women, Fukushima Medical University, 1 Hikarigaoka, Fukushima, 960-1295 Japan; 6 Graduate School of Medical Sciences, Department of Occupational and Environmental Health, Nagoya City University, 1 Kawasumi, Mizuho-cho, Mizuho-ku, Nagoya, Aichi, 467-8601 Japan; 7 National Institute for Environmental Studies, Tsukuba, Japan; 8 National Center for Child Health and Development, Tokyo, Japan; 9 Hokkaido University, Sapporo, Japan; 10 Tohoku University, Sendai, Japan; 11 Chiba University, Chiba, Japan; 12 Yokohama City University, Yokohama, Japan; 13 University of Yamanashi, Chuo, Japan; 14 University of Toyama, Toyama, Japan; 15 Kyoto University, Kyoto, Japan; 16 Osaka University, Suita, Japan; 17 Hyogo College of Medicine, Nishinomiya, Japan; 18 Tottori University, Yonago, Japan; 19 Kochi University, Nankoku, Japan; 20 University of Occupational and Environmental Health, Kitakyushu, Japan; 21 Kumamoto University, Kumamoto, Japan

**Keywords:** Diseases, Health care, Medical research, Risk factors

## Abstract

A high maternal body mass index (BMI) is associated with increased risks of asphyxia-related neonatal morbidity. We evaluated the association between maternal pre-pregnancy BMI and foetal acidosis while accounting for the mode of delivery. Participants from the Japan Environment and Children’s Study with singleton pregnancies after 22 weeks of gestation who gave birth during 2011–2014 were included. The participants (n = 71,799) were categorised into five groups according to the pre-pregnancy BMI: G1 (BMI < 18.5 kg/m^2^), G2 (18.5 to < 20.0 kg/m^2^), G3 (20.0 to < 23.0 kg/m^2^), G4 (23.0 to < 25.0 kg/m^2^), and G5 (≥ 25.0 kg/m^2^). Foetal acidosis was defined as umbilical artery pH (UmA-pH) < 7.20 or < 7.10. Multiple logistic regression analyses were used to evaluate the effect of pre-pregnancy BMI on foetal acidosis risk, accounting for the mode of delivery. In Japanese women, pre-pregnancy BMI ≥ 25.0 kg/m^2^ significantly increased the likelihood of foetal acidosis in newborns delivered vaginally. We found no association between pre-pregnancy BMI and foetal acidosis in newborns delivered via caesarean section. Counselling for body weight control before pregnancy and adequate management and selection of the mode of delivery in pregnant women with a high BMI who are in labour may be essential to avoid foetal acidosis.

## Introduction

Increasing obesity among women in their reproductive years is a growing concern^1^ (overweight is defined as a body mass index [BMI] ≥ 25 kg/m^2^, and obesity as a BMI ≥ 30 kg/m^2^)^2^. In the United States, 37% of women aged 20–39 years are diagnosed with obesity^2,3^, and 48% of women have a BMI ≥ 25 kg/m^2^ at the start of their pregnancies^4^; in Japan, 11% of women have a BMI of ≥ 25 kg/m^2^ at the start of their pregnancies^5^. Maternal obesity increases the risk of pregnancy complications, e.g., gestational diabetes, hypertensive disorders of pregnancy, stillbirths, and preterm births^6–9^. Moreover, a high maternal BMI is associated with increased risks of asphyxia-related neonatal morbidity^10^ and increased incidence of cerebral palsy, neuronal injury, long-term morbidity, and death^11–14^.

Foetal acidosis is a factor associated with birth asphyxia resulting from an interruption of the placental blood flow and subsequent foetal hypoxia and hypercarbia^15–17^. Although several parameters assess neonatal condition, low umbilical artery pH (UmA-pH) has been significantly associated with neonatal mortality^16^, and metabolic acidosis has been associated with foetal hypoxic-ischaemic brain injury^18^. Thus, intrapartum foetal assessments have been refined to increase the detection and thus, reduce the incidence of foetal acidosis^19^.

Using data from the Japan Environment and Children’s Study (JECS), we have reported that excessive gestational weight gain is significantly associated with increased foetal acidosis in women with a pre-pregnancy BMI of 23.0–25.0 kg/m^2^ and not in women with a pre-pregnancy BMI ≥ 25.0 kg/m^2^^20^. We have concluded that in women with a BMI ≥ 25.0 kg/m^2^, the initial bodyweight had a greater impact on foetal acidosis than the gestational weight gain^20^. However, previous studies reporting that maternal obesity increases the risk of foetal acidosis were limited to hospital-based or retrospective cohort study designs and included only vaginal^21,22^ or caesarean^23^ deliveries, not both. As the mode of delivery can affect the neonatal condition^24^, the association between maternal obesity and foetal acidosis should be evaluated while accounting for the mode of delivery.

This study aimed to evaluate the association between maternal pre-pregnancy BMI and foetal acidosis while accounting for the mode of delivery in a large Japanese cohort.

## Results

**Figure 1:**
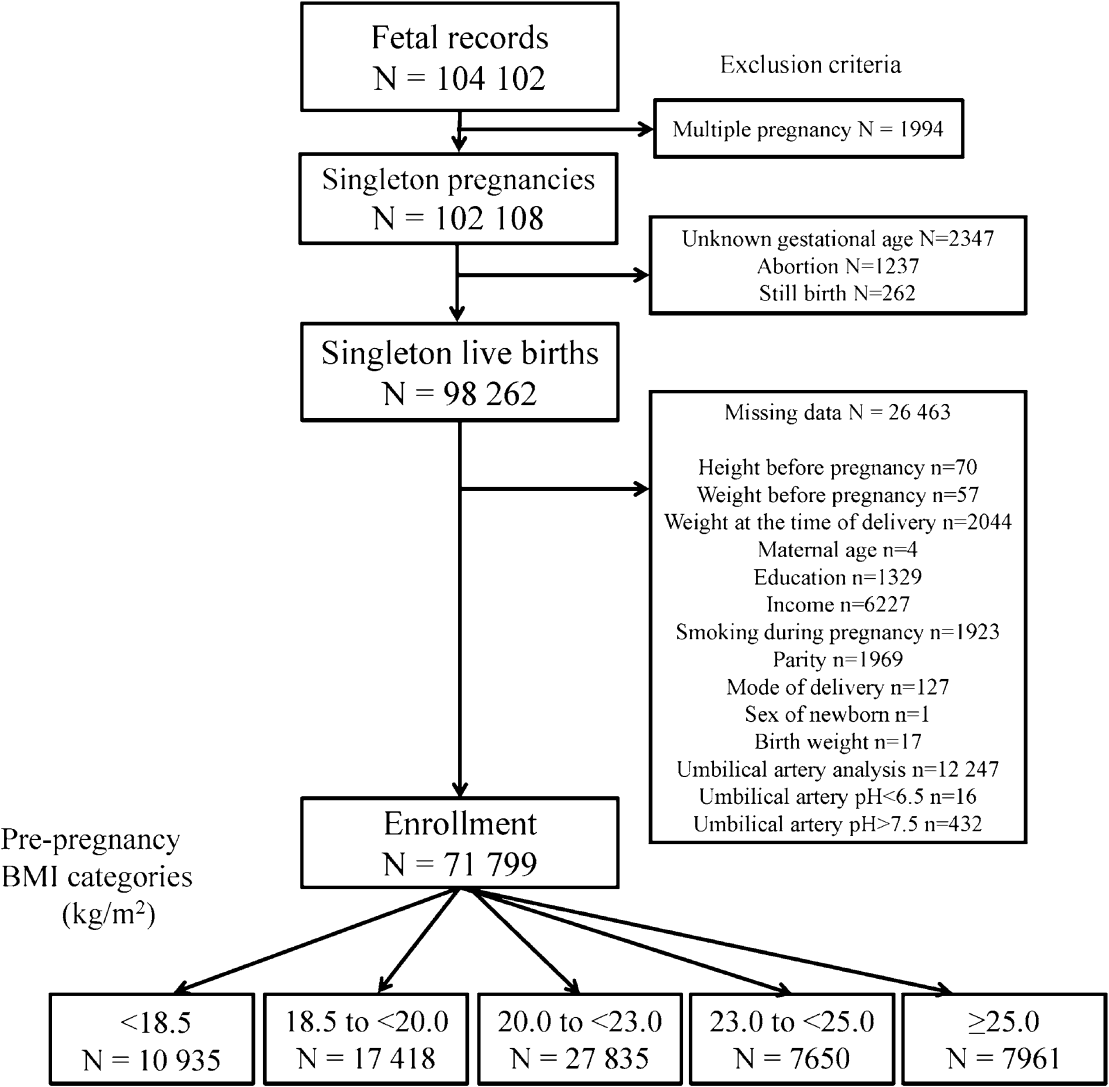
Flowchart of study enrolment.

The total number of foetal records during 2011–2014 was 104,102. After applying our inclusion criteria, 71,799 participants were eligible for the analysis (Fig. 1). There were 10,935 (15.2%) births in G1 (BMI < 18.5 kg/m^2^), 17,418 (24.3%) in G2 (18.5 to < 20.0 kg/m^2^), 27,835 (38.8%) in G3 (20.0 to < 23.0 kg/m^2^), 7650 (10.7%) in G4 (23.0 to < 25.0 kg/m^2^), and 7961 (11.1%) in G5 (≥ 25.0 kg/m^2^) groups. There were 58,345 (81.3%) vaginal deliveries and 13,454 (18.7%) caesarean deliveries.

Table 1 shows the maternal characteristics by pre-pregnancy BMI. Obstetric outcomes by pre-pregnancy BMI are also given in Table 1. G5 had the highest frequencies of caesarean delivery (29.3%), preterm birth (PTB) (5.9%), UmA-pH < 7.20 (7.0%), and UmA-pH < 7.10 (1.3%) as well as the highest average birth weight (3125 g).

**Table 1:** Maternal characteristics and obstetric outcomes by pre-pregnancy BMI.

Maternal characteristics	BMI category (kg/m^2^)					*P*
	G1	G2	G3	G4	G5	
	< 18.5	18.5 to < 20.0	20 to < 23.0	23.0 to < 25.0	≥ 25.0	
	n = 10,935	n = 17,418	n = 27,835	n = 7650	n = 7961	
**Maternal age, %**						
< 20 years	0.9	0.7	0.5	0.3	0.4	< 0.001
20 to 29 years	41.9	36.8	34.6	31.9	31.9	
> 30 years	57.2	62.6	64.9	67.8	67.7	
**Maternal education, %**						
< 10 years	5.1	3.8	3.9	4.4	6.7	< 0.001
10 to 12 years	30.9	28.5	29.8	33.7	39.3	
13 to 16 years	62.5	66.1	64.8	60.8	53.0	
> 17 years	1.5	1.6	1.4	1.2	1.0	
**Annual household income, %**						
< 2,000,000 JPY	6.0	5.0	5.1	6.0	8.2	< 0.001
2,000,000–5,999,999 JPY	67.3	65.6	67.3	69.7	70.9	
6,000,000–9,999,999 JPY	22.1	24.3	23.3	21.2	17.8	
> 10,000,000 JPY	4.6	5.0	4.4	3.0	3.1	
Smoking during pregnancy, %	5.0	3.9	4.1	4.9	6.8	< 0.001
Nulliparous, %	43.4	42.6	39.4	36.6	34.3	< 0.001
Caesarean delivery, %	14.8	15.3	18.4	22.3	29.3	< 0.001
**Obstetric outcomes**						
Gestational age, weeks (mean ± SD)	38.7 ± 1.5	38.9 ± 1.4	38.9 ± 1.5	38.9 ± 1.5	38.8 ± 1.7	< 0.001
PTB before 37 weeks, %	5.0	4.0	4.0	4.2	5.9	< 0.001
Birth weight, g (mean ± SD)	2925 ± 388	2997 ± 387	3049 ± 401	3095 ± 421	3125 ± 467	< 0.001
SGA infants, %	7.3	5.4	4.4	3.8	3.8	< 0.001
UmA-pH < 7.20, %	6.2	5.9	6.4	6.4	7.0	0.014
UmA-pH < 7.10, %	1.1	1.1	1.2	1.2	1.3	0.355

One-way analysis of variance, Kruskal–Wallis test and the chi-square test were used to compare continuous and categorical variables, respectively.

BMI, body mass index; JPY, Japanese yen; LBW, low birth weight; SGA, small for gestational age; SD, standard deviation; UmA-pH, umbilical artery pH.

Table 2 shows the adjusted odds ratios (aORs) for foetal acidosis by pre-pregnancy BMI. The aORs of UmA-pH < 7.20 in G5 were 1.14 (95% confidence interval [CI], 1.03–1.26) in Model 1 and 2, and 1.17 (95% CI, 1.06–1.30) in Model 3, using G3 as the reference group. The aORs of UmA-pH < 7.20 in G2 were 0.90 (95% CI, 0.83–0.98) in Model 1, 0.90 (95% CI, 0.83–0.94) in Model 2, and 0.89 (95% CI, 0.82–0.97) in Model 3, using G3 as the reference group.

**Table 2:** aORs and 95% CIs for intrapartum foetal acidosis by pre-pregnancy BMI.

	BMI category (kg/m^2^)				
	G1	G2	G3	G4	G5
	< 18.5	18.5 to < 20.0	20 to < 23.0	23.0 to < 25.0	≥ 25.0
	n = 10,935	n = 17,418	n = 27,835	n = 7650	n = 7961
**Model with UmA-pH < 7.20**					
Model 1, aOR (95% CI)	0.96 (0.88–1.06)	0.90 (0.83–0.98)	Ref	1.02 (0.92–1.13)	1.14 (1.03–1.26)
Model 2, aOR (95% CI)	0.95 (0.87–1.04)	0.90 (0.83–0.94)	Ref	1.02 (0.92–1.14)	1.14 (1.03–1.26)
Model 3, aOR (95% CI)	0.94 (0.86–1.03)	0.89 (0.82–0.97)	Ref	1.03 (0.93–1.15)	1.17 (1.06–1.30)
**Model with UmA-pH < 7.10**					
Model 1, aOR (95% CI)	0.87 (0.70–1.07)	0.86 (0.71–1.02)	Ref	0.99 (0.78–1.25)	1.10 (0.88–1.38)
Model 2, aOR (95% CI)	0.84 (0.68–1.04)	0.85 (0.71–1.02)	Ref	0.99 (0.78–1.25)	1.09 (0.87–1.36)
Model 3, aOR (95% CI)	0.85 (0.69–1.05)	0.85 (0.71–1.02)	Ref	0.98 (0.78–1.24)	1.07 (0.85–1.33)

Logistic regression models were used to calculate the adjusted odds ratios and 95% CIs for UmA-pH < 7.20 and < 7.10, using G3 as the reference group.

aOR, adjusted odds ratio; BMI, body mass index; CI, confidence interval; Ref., reference; UmA-pH, umbilical artery pH.

Model 1 was adjusted for maternal age, maternal education, annual household income, maternal smoking during pregnancy, and parity.

Model 2 was adjusted for covariates in model 1 and preterm birth and small for gestational age infants.

Model 3 was adjusted for covariates in model 2 and mode of delivery.

Table 3 shows the aORs for foetal acidosis after stratification of the participants by mode of delivery. In the vaginal delivery group, the aOR of UmA-pH < 7.20 in G5 was 1.12 (95% CI, 1.08–1.35); that of UmA-pH < 7.20 in G2 was 0.90 (95% CI, 0.83–0.98); and that of UmA-pH < 7.10 in G2 was 0.80 (95% CI, 0.65–0.98), using G3 as the reference group. In the caesarean delivery group, there was no association between pre-pregnancy BMI and foetal acidosis.

**Table 3:** aORs and 95% CIs for intrapartum foetal acidosis by pre-pregnancy BMI after stratification based on mode of delivery.

Mode of delivery	BMI category (kg/m^2^)				
	G1	G2	G3	G4	G5
	< 18.5	18.5 to < 20.0	20 to < 23.0	23.0 to < 25.0	≥ 25.0
**For UmA-pH < 7.20**					
Vaginal delivery (n = 58,345)	n = 9321	n = 14,752	n = 22,703	n = 5942	n = 5627
aOR (95% CI)	0.93 (0.84–1.02)	0.90 (0.83–0.98)	Ref	1.03 (0.91–1.15)	1.12 (1.08–1.35)
Caesarean section (n = 13,454)	n = 1614	n = 2666	n = 5132	n = 1708	n = 2334
aOR (95% CI)	1.04 (0.81–1.33)	0.82 (0.66–1.02)	Ref	1.07 (0.84–1.36)	1.06 (0.86–1.32)
**For UmA-pH < 7.10**					
Vaginal delivery (n = 58,345)	n = 9321	n = 14,752	n = 22,703	n = 5942	n = 5627
aOR (95% CI)	0.79 (0.63–1.01)	0.80 (0.65–0.98)	Ref	0.92 (0.70–1.22)	1.12 (0.86–1.46)
Caesarean section (n = 13,454)	n = 1614	n = 2666	n = 5132	n = 1708	n = 2334
aOR (95% CI)	1.10 (0.70–1.75)	1.10 (0.74–1.62)	Ref	1.20 (0.77–1.89)	1.01 (0.66–1.55)

Logistic regression models were used to calculate the adjusted odds ratios and 95% CIs for UmA-pH < 7.20 and < 7.10, using G3 as the reference group.

aOR, adjusted odds ratio; BMI, body mass index; CI, confidence interval; Ref., reference; UmA-pH, umbilical artery pH.

Model adjusted for maternal age, maternal education, annual household income, maternal smoking during pregnancy, parity, preterm birth, and small for gestational age infants.

## Discussion

This study evaluated the association between maternal pre-pregnancy BMI and foetal acidosis while accounting for the mode of delivery. In this cohort, a pre-pregnancy BMI ≥ 25.0 kg/m^2^ increased the likelihood of foetal acidosis in newborns delivered vaginally, but not in those delivered by caesarean section.

Our finding that high maternal BMI was associated with foetal acidosis is consistent with that reported in recent studies^10,22^, although we used a different categorisation previously described for the Japanese women, which defined obesity as BMI ≥ 25 kg/m^2^^25^. This categorisation was also validated by the World Health Organization’s suggestion that a modified BMI threshold ≥ 23 kg/m^2^ rather than ≥ 25 kg/m^2^ should be used to define the overweight condition for Asians^26^. The result that a pre-pregnancy BMI ≥ 25.0 kg/m^2^ significantly increased foetal acidosis suggests that Japanese-defined obesity, which is comparable to overweight and obesity worldwide^2,27^, is a significant risk factor for foetal acidosis. In this study, the risk significantly increased at UmA-pH < 7.20, but not at UmA-pH < 7.10. This might have been because the study population size for UmA-pH < 7.20 and < 7.10 decreased according to the progress of foetal acidosis. While births with UmA-pH < 7.20 accounted for 6.3% of all births in the study population, births with UmA-pH < 7.10 accounted for only 1.2%. In Japan, rapid intervention, such as emergent caesarean section and intrauterine foetal resuscitation, to reduce foetal acidosis seems to decrease its incidence; therefore, information regarding births with UmA-pH < 7.20 would be important for adequate perinatal care.

High maternal pre-pregnancy BMI was reported as an independent predictor of birth asphyxia in mothers without diabetes or preeclampsia^10^. Maternal obesity is associated with insulin resistance in the offspring of mothers without diabetes^28^ and foetal hyperinsulinemia^29^, which may cause chronic foetal hypoxia. Moreover, maternal chronic inflammation and oxidative stress caused by obesity may cause placental inflammation and placental insufficiency, resulting in foetal hypoxia^30,31^.

Low maternal BMI was associated with lower risks for foetal acidosis. To our knowledge, previous studies have not reported an association between lean women and foetal acidosis. Lean pregnant women have shorter first and second stages of labour than their normal-weight counterparts^32^, which may decrease the risk of foetal acidosis. We found that lower maternal pre-pregnancy BMI could reduce foetal acidosis. However, as lean women may experience adverse obstetric outcomes, e.g. low birth weight and premature birth^26^, we do not suggest that pregnant women should be lean.

The strength of this study is that we accounted for the mode of delivery, contrary to previous studies^21–23^. Our finding that vaginal delivery in women with BMI ≥ 25.0 kg/m^2^ was associated with foetal acidosis is consistent with that found in recent studies^21,22^. Regarding vaginal deliveries, the increased risk of foetal acidosis in women with high BMI may be associated with shoulder dystocia^33^ or traumatic labour due to foetal macrosomia, frequently observed in obese women^34^. Our large cohort study strengthened the same result suggested by previous smaller studies^21,22^.

Our finding that caesarean delivery in women with a pre-pregnancy BMI ≥ 25.0 kg/m^2^ was not associated with foetal acidosis is not consistent with a recent study’s result^23^. This previous study reported that umbilical cord pH decreased as maternal BMI increased in caesarean section deliveries^23^. Additionally, increased maternal BMI may increase operative difficulties or affect maternal haemodynamic or pulmonary function intraoperatively, decreasing maternal oxygenation and placental perfusion^23^. The discrepancy of the result may have resulted from the difference in the proportion of enrolled participants. Here, 44 of 13,454 (0.3%) participants had a pre-pregnancy BMI ≥ 40 kg/m^2^ compared to 733 of 5488 (13.4%) participants reported previously^23^. This may have increased the number of cases with operative difficulties in the past study. The Japanese population, which has fewer obese pregnant women than the worldwide population^4,5^, may show less risks of foetal acidosis in cases of caesarean section. In caesarean section cases, the absence of uterine contractions during labour causes a decreased incidence of umbilical compression leading to foetal hypoxia. Obstetricians should consider the mode of delivery to best incorporate maternal and foetal risks and benefits.

This study has several limitations. First, regarding the mode of delivery, data about assisted vaginal delivery and detailed data about caesarean section were lacking. Since the present data set had insufficient data about assisted vaginal delivery with vacuum or forceps regarding the indication and the difficulties, the present analysis did not consider the difference of spontaneous delivery and assisted vaginal delivery. Careful interpretation is required because assisted vaginal delivery would carry higher risks for foetal acidosis. Moreover, data about the indication of caesarean section, elective caesarean section or emergent caesarean section, or vaginal delivery trial were lacking. These data may directly affect the foetal condition; thus, further studies are needed to clarify the effect of the background and the pattern of caesarean section. Second, the mechanism of foetal acidosis was not considered. Foetal acidosis may be caused by a respiratory or metabolic mechanism, and UmA-pH does not discriminate between them^35^. The identification of metabolic acidosis is a key criterion for establishing a causal relationship between foetal perinatal asphyxia and neonatal encephalopathy and/or cerebral palsy^18,36^, because purely respiratory acidosis is not associated with neonatal adverse outcomes^36,37^. Here, foetal acidosis included foetal respiratory acidosis. Therefore, careful interpretation of our findings and further study of the mechanism underlying the association between maternal BMI and foetal acidosis are needed. Third, the present analysis could not expand the analysis to include severe birth-asphyxia-related complications, e.g. meconium aspiration syndrome, because this kind of information was lacking in the present data set. Therefore, we could not determine if the increased rate of foetal acidosis was related to severe birth-asphyxia-related complications. However, the JECS is a nationwide prospective birth cohort study, and long-term neonatal and offspring outcomes can be analysed in the future. Further studies may clarify the effect of pre-pregnancy BMI on long-term neonatal and offspring outcomes in the JECS.

In conclusion, we showed that increased maternal pre-pregnancy BMI was significantly associated with foetal acidosis in Japanese women, particularly those undergoing vaginal delivery. Therefore, preconception counselling for body weight control and adequate management and selection of mode of delivery in pregnant women with pre-pregnancy BMI ≥ 25.0 kg/m^2^ in labour may be essential to avoid foetal acidosis.

## Methods

### Study design

We used data derived from the JECS, which is a nationwide prospective birth cohort study established in January 2011 to investigate the effects of environmental factors on children’s health^38,39^. Briefly, the JECS is funded directly by Ministry of the Environment, Japan and involves collaboration between the Programme Office (National Institute for Environmental Studies), the Medical Support Centre (National Centre for Child Health and Development), and 15 regional centres (Hokkaido, Miyagi, Fukushima, Chiba, Kanagawa, Koshin, Toyama, Aichi, Kyoto, Osaka, Hyogo, Tottori, Kochi, Fukuoka, and South Kyushu/Okinawa)^39^. The eligibility criteria for the JECS participants (expectant mothers) were as follows: (1) residence in the study areas at the time of recruitment and the expectation to continually reside in Japan for the foreseeable future; (2) an expected delivery date between August 1, 2011, and mid 2014; and (3) capability to participate in the study without difficulty (i.e. ability to comprehend the Japanese language and complete the self-administered questionnaire).

Either or both of the following two recruitment protocols were applied: (1) recruitment at the time of the first prenatal examination at cooperating obstetric facilities; and (2) recruitment at local government offices issuing a pregnancy journal, called the Maternal and Child Health Handbook, that is given to all expectant mothers in Japan before they receive municipal services for pregnancy, delivery, and childcare. We contacted pregnant women via cooperating health care providers and/or local government offices issuing Maternal and Child Health Handbooks and registered those willing to participate. Self-administered questionnaires, which were completed by the women during the first trimester and second/third trimester, were used to collect information on demographic factors, medical and obstetric history, physical and mental health, lifestyle, occupation, environmental exposure at home and in the workplace, housing conditions, and socioeconomic status^39^.

The JECS protocol and the present study were reviewed and approved by the Ministry of the Environment Institutional Review Board on Epidemiological Studies and by the ethics committees of all participating institutions (Independent Ethics Committee [IEC] of the National Center for Child Health and Development, Hokkaido University, Institutional Review Board [IRB] of Sapporo Medical University, IEC of the Asahikawa Medical College, IEC of the Japanese Red Cross Hokkaido College of Nursing, IEC of Tohoku University, IEC of Fukushima Medical University, IRB of Chiba University, IEC of Yokohama City University, IEC of the University of Yamanashi, IEC of Shinshu University, The Ethics Committee of Toyama University, IRB of Nagoya City University, IEC of Kyoto University, The Doshisha University Research Ethics Review Committee Regarding Human Subject Research, IEC of Osaka University, IEC of Osaka Medical Center and Research Institute for Maternal and Child Health, IEC of Hyogo College of Medicine, IRB of Tottori University, The Research Ethics Committee of Kochi University, IRB of The University of Occupational and Environmental University [the origination of this study], IEC of Kyushu University, IEC of Kumamoto University, IEC of the University of Miyazaki, and IEC of the University of the Ryukyus). The JECS was conducted in accordance with the Helsinki Declaration and other national regulations and guidelines. All methods of this study were carried out in accordance with relevant guidelines and regulations. Written informed consent was obtained from all participants.

### Data collection

The current analysis used the data set released in June 2016 (data set: jecs-ag-20160424). Specifically, we used three types of data: (1) M-T1, obtained from a self-reported questionnaire that was collected during the first trimester (the first questionnaire) and that included questions regarding maternal medical background; (2) M-T2, obtained from a self-reported questionnaire that was collected during the second or third trimester (second questionnaire) and that included socioeconomic status; and (3) Dr-0m, collected from medical records provided by each subject’s institution, that included obstetrical outcomes such as gestational age, birth weight, and UmA-pH.

Participants with singleton pregnancies after 22 weeks were included in the present study. Women with multiple pregnancies, abortion, still births, and missing information were excluded from the analysis. There were no significant differences in patient characteristics between those included and excluded (data not shown).

### Exposure variables, obstetric outcomes, and confounding factors

Pre-pregnancy BMI was calculated according to the WHO standards (bodyweight [kg]/height^2^ [m^2^]). The participants were categorised into five groups according to their pre-pregnancy BMI: G1 (BMI < 18.5 kg/m^2^), G2 (18.5 to < 20.0 kg/m^2^), G3 (20.0 to < 23.0 kg/m^2^), G4 (23.0 to < 25.0 kg/m^2^), and G5 (≥ 25.0 kg/m^2^). These categories were based on the criteria for Japanese women proposed by Morisaki et al. to predict adverse obstetric outcomes based on pre-pregnancy BMI^20,25^. In Japan, obesity during pregnancy is defined as BMI ≥ 25.0 kg/m^2^ (G5)^25^, which is comparable to overweight and obesity in western countries^2,27^.

Foetal acidosis was defined as UmA-pH < 7.20 or < 7.10 from Dr-0m data. These thresholds were chosen based on a study reporting that UmA-pH < 7.20 increased the risk for short-term neonatal adverse outcomes^40^ and another one reporting that UmA-pH < 7.10 increased the risk for neonatal adverse neurological sequelae^17^. We did not analyse the data regarding women with UmA-pH < 7.00 because the number of patients in this group was too small (0.2%).

The following factors were considered as potential confounders: maternal age, maternal education, annual household income, maternal smoking during pregnancy, parity, PTB, small for gestational age (SGA) infants, and mode of delivery. Maternal age was categorised into three age groups: < 20, 20 to 29, and ≥ 30 years based on a previous study reporting that maternal age was associated with certain obstetric outcomes, such as PTB, low birth weight infants, and SGA infants^41,42^. Maternal educational level was categorised into four groups: junior high school (< 10 years of education), high school (10–12 years of education), professional school or university (13–16 years of education), and graduate school (≥ 17 years of education). Annual household income was categorised into four levels: < 2,000,000, 2,000,000–5,999,999, 6,000,000–9,999,999, and ≥ 10,000,000 Japanese yens. Participants were requested to provide information about their smoking status by choosing one of the following options: ‘kept smoking during pregnancy’, ‘never smoked’, ‘quit smoking before pregnancy’, and ‘quit smoking during early pregnancy’. Participants who chose ‘kept smoking during pregnancy’ comprised the smoking category, while the other participants comprised the non-smoking category. Parity obtained from M-T1 data was categorised into nulliparous or multiparous. PTB was defined as delivery before 37 weeks. SGA was defined as birth weight < -1.5 standard deviations corrected for gestational age and sex according to the new Japanese neonatal anthropometric charts for gestational age at birth^43^. The mode of delivery was categorised into vaginal or caesarean delivery from medical records. These confounding factors were chosen based on clinical importance.

### Statistical analysis

Maternal characteristics were summarised based on pre-pregnancy BMI. One-way analysis of variance, Kruskal–Wallis test and the chi-square test were used to compare continuous and categorical variables, respectively. Logistic regression models were used to calculate the aORs and 95% CIs for UmA-pH < 7.20 and < 7.10, using G3 as the reference group. In Model 1, maternal age, maternal education, annual household income, maternal smoking during pregnancy, and parity were considered as confounding factors to calculate the aORs of the effect of pre-pregnancy BMI on foetal acidosis. In Model 2, PTB and delivery of SGA infants were added as confounding factors (in addition to those of Model 1) to calculate aORs for foetal acidosis. In Model 3, mode of delivery was added as a confounding factor (in addition to those of Model 2) to calculate aORs for foetal acidosis. After this analysis, we stratified the participants based on the mode of delivery, and logistic regression models were used to calculate aORs and 95% CIs for foetal acidosis using the confounding factors of Model 2.

SPSS version 26 (IBM Corp., Armonk, NY, USA) was used for the statistical analysis. A *P* value < 0.05 was considered to indicate statistical significance.

## Data Availability

Data are unsuitable for public deposition due to ethical restrictions and legal framework of Japan. It is prohibited by the Act on the Protection of Personal Information (Act No. 57 of 30 May 2003, amendment on 9 September 2015) to publicly deposit the data containing personal information. Ethical Guidelines for Epidemiological Research enforced by the Japan Ministry of Education, Culture, Sports, Science and Technology and the Ministry of Health, Labour and Welfare also restricts the open sharing of the epidemiologic data. All inquiries about access to data should be sent to: jecs-en@nies.go.jp. The person responsible for handling enquiries sent to this e-mail address is Dr. Shoji F. Nakayama, JECS Programme Office, National Institute for Environmental Studies.
